# Repetitions of Strenuous Exercise Consistently Increase Paraoxonase 1 Concentration and Activity in Plasma of Average-Trained Men

**DOI:** 10.1155/2021/2775025

**Published:** 2021-12-07

**Authors:** Aneta Otocka-Kmiecik, Monika Orłowska-Majdak, Robert Stawski, Urszula Szkudlarek, Piotr Kosielski, Gianluca Padula, Szymon Gałczyński, Dariusz Nowak

**Affiliations:** ^1^Department of Experimental Physiology, Interfaculty Chair of Experimental and Clinical Physiology, Medical University of Lodz, 6/8 Mazowiecka St., 92-215 Lodz, Poland; ^2^Department of Clinical Physiology, Interfaculty Chair of Experimental and Clinical Physiology, Medical University of Lodz, 6/8 Mazowiecka St., 92-215 Lodz, Poland; ^3^Academic Laboratory of Movement and Human Physical Performance, Medical University of Lodz, 251 Pomorska St., 92-213 Lodz, Poland

## Abstract

**Objectives:**

Oxidative stress, induced by physical activity, may stimulate the expression, release, and activity of certain antioxidant enzymes. We investigated the effect of three repeated bouts of strenuous exercise on paraoxonase 1 concentration (PON1c) and paraoxonase activity (PON).

**Methods:**

Eleven average-trained healthy men (age 34.0 ± 5.2 years) performed three strenuous exercise tests on a treadmill separated by 72 hours periods of resting. PON1c, PON, ferric-reducing activity of plasma (FRAP), lipid profile, C-reactive protein concentration (CRP), and lactate concentration were determined in plasma.

**Results:**

Each exercise bout resulted in similar PON1c, PON, FRAP, and high-density lipoprotein concentration (HDL-C) increments, while PON/HDL-C ratio remained stable in all repetitions. Percentage increments at the bout of each exercise were higher for PON1c (by 64.82% at the first, by 92.9% at the second, and by 77.02% at the third exercise) than for PON (by 6.49% at the first, 10.06% at the second, and by 12.32% at the third exercise). Association was found between preexercise PON and PON1c (*r* = 0.56, *p* = 0.029), pre- (*r* = 0.87, *p* = 0.00003) and postexercise HDL-C (*r* = 0.6, *p* = 0.0002), preexercise PON and cardiovascular fitness level of participants measured as VO_2_max (*r* = 0.39, *p* = 0.026), and postexercise PON and lactate concentration (*r* = 0.44, *p* = 0.01).

**Conclusions:**

PON1c and PON increase during strenuous exercise, yet the effect of exercise on PON1 concentration is more pronounced. PON1 does not show tolerance to physical activity. The enzyme may provide short-term protection from oxidative stress in each exercise bout. PON may depend on exercise load. Cardiovascular fitness levels may be associated with PON1 activity.

## 1. Introduction

There is substantial evidence proving that a single strenuous exercise induces oxidative stress. As oxygen consumption increases up to 20-fold during endurance exercise, the production of reactive oxygen species (ROS) exceeds the body's ability to detoxify them. In high concentrations, ROS produce adverse modifications to cell components, such as lipids, proteins, and DNA [1, 2]. However, regular physical activity is a known protective factor against cardiovascular disease. Therefore, it seems reasonable that in the case of regularly repeated endurance exercise, defensive mechanisms must be developed in human plasma, which fight the deleterious effects of oxidative stress. Those mechanisms involve various antioxidants, among which PON1 may play an important role.

PON1 is an enzyme, which is of special significance in relation to atherosclerosis prevention, as it is attached to HDL (high-density lipoprotein) and protects LDL (low-density lipoprotein) from oxidation. In macrophages, PON1 reduces total peroxide levels and superoxide production [3]. Some evidence shows that PON1 directly binds to the atherosclerotic plaque and suppresses foam cell formation [4]. It has lactonase activity, hydrolyzes homocysteine thiolactone, and protects against the accumulation of N-homocysteine protein [5].

The assessment of PON1 status involves not only nucleotide polymorphism but also PON1 concentration and paraoxonase (PON) activity, i.e., activity against an artificial substrate, paraoxon. In humans, the PON1 gene is localized between q21 and q22 on the long arm of chromosome 7 [6]. There are two common polymorphisms in the coding region of the gene: leucine (L) to methionine (M) substitution at position 55 (L55M) and glutamine (Q) to arginine (R) substitution at position 192 (Q192R) [7]. The resulting isoenzymes have different hydrolytic activities towards paraoxon, i.e., R and L alleles have higher activities than their respective allele isoforms, Q and M [8]. Yet, it is the Q and M allele isoforms that are more effective in protecting LDL molecules against oxidative modification than R and L isoforms [8].

PON1 activity is known to be dependent, not only on genetic but also lifestyle and environmental factors such as physical activity [9]. Our previous research shows that maximal exercise leads to a significant increase in PON1 activities in the plasma of young men [10–12]. This increase is followed by a decrease or at least a return, to the basal levels in an hour and two hours after exercise.

It was also found that training, mainly in sportsmen, consolidates the changes in PON1 activity. Martinovic et al. observed that PON1's ability to resist constantly repeated bouts of ROS release is enhanced by regular physical activity, as 8–10 years of training resulted in an increase in PON1 activity to maximal levels [13]. Arslan et al. observed that PON activity was higher in a group, which undertook regular exercise than in the acute exercise group. The sedentary group proved to have even lower PON activity [14].

However, the effect of repetition of the same exercise bouts on PON1 level has not been studied so far. We speculate that with subsequent repetitions of exercise, there may be occurring some changes affecting the PON1 enzyme, which finally may lead to increased PON1 activity or/and concentration in regularly training subjects during physical activity and at rest.

Therefore, the present study is aimed at investigating and comparing the effect of three repeated bouts of exercise on PON1 activity and concentration. By repeating the exercise three times, we wanted to test the hypothesis that subsequent repetitions of strenuous physical activity may lead to a different change in antioxidant defence provided by PON1 through development of tolerance to exercise. Moreover, associations between PON1 concentration, activity, and chosen parameters measured at exercise were examined. The findings of the present study may provide an experimental proof about the endurance training adaptations of antioxidant enzymes such as PON1.

## 2. Materials and Methods

### 2.1. Subjects

A group of 11 healthy, average-trained men aged 34.0 ± 5.2 years with a training experience of 10-15 years was enrolled in the study. Their training program consisted of an average of 1 hour daily aerobic exercise sessions performed three times a week and 1.5-hour long soccer match once a week. The participants were nonsmokers and were not taking any drugs known to affect lipid metabolism or antioxidant capacity of plasma. Informed consent was signed by the volunteers upon participation in the experiment.

### 2.2. Study Protocol

The study protocol was described in detail in our previous report [15]. Briefly, the study consisted of four visits (on observation days 1, 7, 10, and 13). They all started at 9 : 00 a.m., included medical examination, resting ECG, and blood pressure measurements. On the first visit (day 1), preliminary testing was conducted, which consisted of spirometry tests and a continuous incremental treadmill run for personal VO_2_max determination. Trackmaster CP 425 treadmill was used, interfaced with a gas exchange analysis system Ultima CardiO2 PFX. Afterward, each subject came for the next three visits with a time interval of 3 days (days 7, 10, and 13), which followed the same protocol. Volunteers performed a treadmill run to volitional exhaustion at a speed corresponding to 70% of their personal VO_2_max. Venous blood samples were collected twice, directly pre- and directly postexercise, into lithium heparin and EDTA Vacutainer tubes (Becton, Dickinson and Company, New Jersey, USA). The blood was centrifuged (3000 × g, 4°C, 15 min), and blood plasma was stored at -80°C for further analysis. Plasma volume shifts caused by dehydration due to sweating and breathing in response to exercise were determined. However, haematocrit increases after exercise bouts were small and did not exceed 1.5%. All results obtained from plasma analysis were corrected for the plasma volume shifts.

During the whole study period (13 days), subjects were asked to keep their dietary patterns stable and to refrain from exhaustive exercises besides those related to the experiment.

All methods were carried out in accordance with the Declaration of Helsinki. The study protocol was approved by the Bioethics Committee of Medical University of Lodz no. RNN/95/14/KB.

### 2.3. Determinations in Plasma

PON1 activity, paraoxonase, marked as PON was measured with its substrate, paraoxon, as described by Nakanishi et al. [16]. Total antioxidant activity was measured as ferric-reducing activity of plasma (FRAP), according to Benzie and Strain [17]. PON1 concentration, marked as PON1c, was measured using ELISA Kit for paraoxonase 1 (USCN Life Sciences, Inc. Wuhan, China). The detection range of the assay was 3.12-200 ng/ml, sensitivity 1.27 ng/ml.

Complete blood count, C-reactive protein concentration (CRP), concentrations of lactate, creatinine, glucose, urea, creatine kinase activity (CK), total cholesterol concentration (TChol), high-density lipoprotein concentration (HDL-C), low-density lipoprotein concentration (LDL-C), and triglyceride concentration (TG) were determined with Bekman Culter Analyser AU680 in the Diagnostic Laboratory of Central Clinical Hospital of the Medical University of Lodz, Poland.

### 2.4. Genotyping of the PON1 Gene

The PON1 single-nucleotide polymorphisms were analysed using TaqMan SNP Genotyping Assays (Applied Biosystems) as described previously [18]. Genomic DNA was extracted from *peripheral blood leukocytes by a QIAamp DNA Blood Mini Kit (Qiagen)* as in the manufacturer's instructions. DNA was quantified using a Picodrop spectrophotometer (Picodrop Limited) and diluted to a concentration of 2 ng/*μ*l. DNA samples were stored in TE buffer (5 mM Tris–HCl, 0.1 mM EDTA, and pH = 8.5) at −20°C until analysis [19]. PCR was performed using the GeneAmp PCR System 9700 (Applied Biosystems) in 20 *μ*l reaction volume containing 10 ng DNA, 10 *μ*l TaqMan® Universal PCR Master Mix, and 0.5 *μ*L (40x) appropriate TagMan SNP Genotyping Assay. For PCR amplification, we followed the protocol in [20]: preincubation at 95°C for 10 min, followed by 40 cycles of denaturation at 92°C for 15 sec, and primer annealing and extension at 60°C for 1 min. Each 96-well plate contained tested samples and 3 blanks. Fluorescent intensities of each probe were monitored using the 7900HT Fast Real-Time PCR System (Applied Biosystems) and captured using the Sequence Detection System 2.3 Software.

The sequences of VIC and FAM probes used for polymorphism detection are shown in Table 1.

PON1c and PON1 genotypes were assessed in The Central Scientific Laboratory of Medical University in Lodz (CoreLab).

### 2.5. Chemicals

Trizma base was derived from Fluka (Buchs, Switzerland), Triton X-100 was from Serva Feinbiochemica (Heidelberg, Germany), and all other reagents were derived from Sigma Aldrich Chemical (St Louis, MO, USA).

### 2.6. Statistical Analysis

The analysis was carried out with Statistica Software v13. Results were expressed as mean ± SD. Data for the three repeated visits were analysed by using ANOVA for repeated observations followed by Scheffe's test or Friedman ANOVA followed by post hoc Wilcoxon matched pairs test depending on the data distribution measured by Shapiro-Wilk's test. Spearman's correlation coefficient (*r*) was estimated to determine the association between the chosen variables. Statistical significance was set at *p* < 0.05.

## 3. Results

The basic physiological characteristics of the subjects during repeated exercise are enclosed in Table 2 (data shown previously [15]). It is worth noting that some changes in these parameters in subsequent repetitions show a tendency for adaptation to repeated exercise, which is evident in the case of increasing running distance and time. At each repetition, participants ran to volitional exhaustion. Yet, already in the 2nd run and then in the 3rd run, we observed an adjustment to this form of exercise and equipment, which resulted in the potential to complete a prolonged treadmill run.

Genotyping of the PON1 gene revealed an uneven allele distribution in the Q192R polymorphism among participants (3 subjects with QQ, 7 subjects with QR, and 1 subject with RR genotype) (see Supplementary Table S5). Genotyping the PON1 gene for the L55M polymorphism revealed 5 subjects with LL, 6 subjects with LM, and no subjects with MM genotype.

### 3.1. The Effect of Three Repeated Bouts of Exercise on PON Activity, PON1c, and Other Biochemical Variables in Plasma

**Figure 1:**
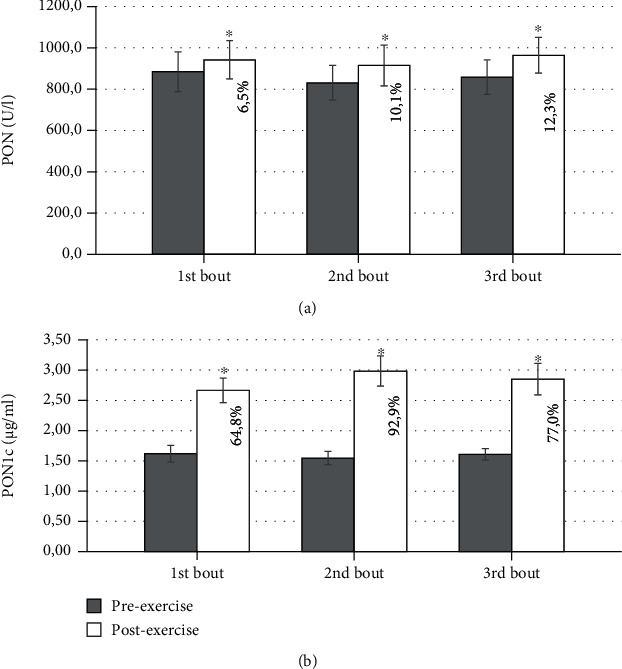
Effect of repeated exercise on: (a) paraoxonase activity (PON) and (b) paraoxonase 1 concentration (PON1c) (mean ± SD). PON1c increment after exercise is shown additionally in percentage values. ^∗^Before vs. bout of exercise, *p* < 0.05.

**Figure 2:**
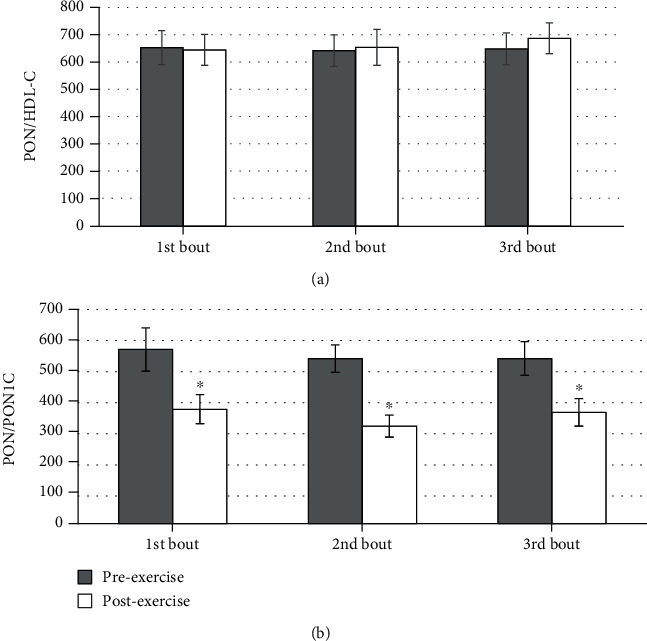
Effect of repeated exercise on (a) paraoxonase to high-density cholesterol concentration ratio (PON/HDL-C) and (b) paraoxonase to paraoxonase 1 concentration ratio (PON/PON1c) (mean ± SD). ^∗^Before vs. bout of exercise, *p* < 0.05.

Table 3, Figures 1 and 2, and Supplementary Tables S6, S7, and S8 illustrate the obtained results (for individual data see Supplementary Tables S1, S2, S3, and S4). In each repetition of the exercise, we found an increase in postexercise PON. Furthermore, each subsequent exercise bout resulted in a higher percentage increment of PON. However, the observed increase in PON was not long-lasting. The exact time of return to the baseline level is not known, as the next observation was scheduled 72 hours after exercise. At that time, PON returned to baseline values. The preexercise PON did not differ in any of the measurements.

PON1c increased at the bout of each exercise. The lowest increment was noted at the first, highest at the second, and intermediate at the third bout of exercise.

The pre- and postexercise absolute levels of PON and PON1c did not change when subsequent visits were compared.

PON standardized for PON1c (PON/PON1c ratio) was higher pre- than postexercise (first and second bout *p* = 0.003 and third bout *p* = 0.016) (Table 3, Figure 2). We did not find differences in this ratio when the three bouts of exercise were compared.

FRAP increased at each bout of exercise. The increment in FRAP was transient as the values returned to baseline levels within 72 hours after the first and the second exercise. There was no difference in FRAP when comparing all three preexercise and postexercise measurements.

An increase in HDL-C at the bout of each session of exercise was observed. There was no difference in preexercise HDL-C in all three measurements. At the same time, HDL-C was significantly higher postexercise at the first exercise in comparison to the second and third repetitions. Therefore, the first exercise resulted in the highest HDL-C increment.

TChol, LDL-C, and TG were significantly higher pre- and postexercise in the first treadmill run in comparison to the second and third ones. These variables tended to increase at the bout of each exercise, although a significant difference was not always reached.

The remaining parameters usually increased at each exercise bout as per a well-known effect of physical activity on muscular metabolism.

### 3.2. Correlation between PON and Other Parameters

As the study was performed on a relatively low number of participants (*n* = 11), the direct analysis of correlations between different variables at each exercise bout was inconclusive. Therefore, to overcome this problem, we pooled the data from three exercise bouts into the following datasets: (A) pooled preexercise individual data (*n* = 33) and (B) pooled postexercise individual data (*n* = 33).

Correlation of PON with aerobic endurance indices showed a positive correlation of moderate strength between preexercise PON and VO_2_max of subjects (*r* = 0.39, *p* = 0.026). Postexercise PON correlated negatively with the running distance (*r* = −0.375, *p* = 0.03) and time of exercise (*r* = −0.37, *p* = 0.036) and positively with lactate postexercise concentration (*r* = 0.44, *p* = 0.01) (Supplementary Fig. S1).

**Figure 3:**
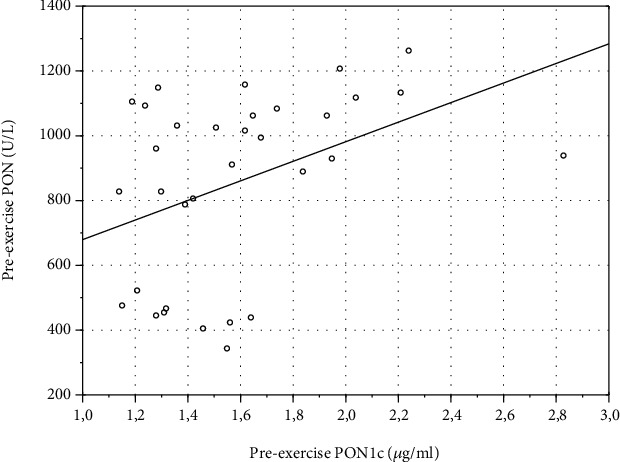
Scatterplot of variables *x* and *y*. Correlation of preexercise values of PON1c and PON in eleven average-trained healthy men. Pooled individual preexercise data from three exercise bouts (*n* = 33; *r* = 0.38, *p* = 0.03).

**Figure 4:**
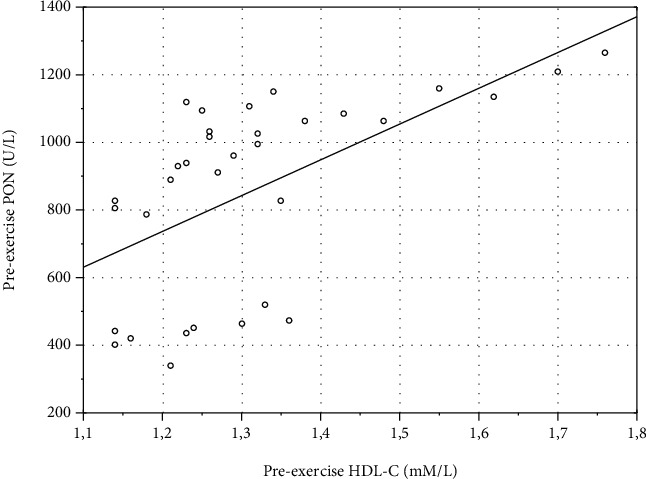
Scatterplot of variables *x* and *y*. Correlation of preexercise values of HDL-C and PON in eleven average-trained healthy men. Pooled individual preexercise data from three exercise bouts (*n* = 33; *r* = 0.68, *p* = 0.00002).

**Figure 5:**
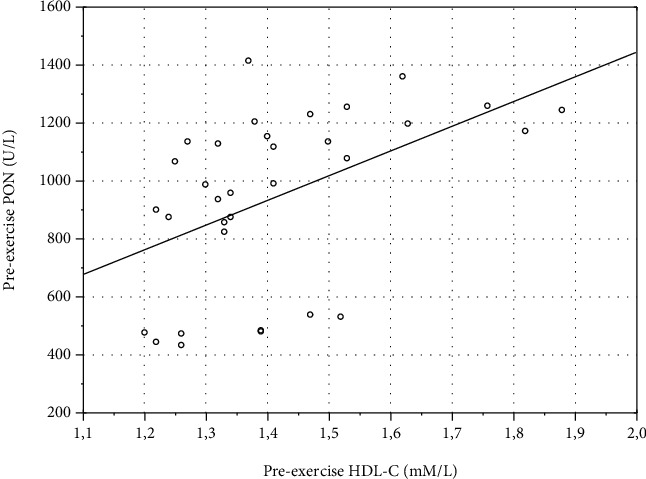
Scatterplot of variables *x* and *y*. Correlation of postexercise values of HDL-C and PON in eleven average-trained healthy men. Pooled individual postexercise data from three exercise bouts (*n* = 33; *r* = 0.6, *p* = 0.0002).

Correlation with blood biochemical data revealed a moderate positive correlation between preexercise values of PON1c and PON (*r* = 0.38, *p* = 0.03) (Figure 3). HDL-C strongly correlated with pre- (*r* = 0.68, *p* = 0.00002) (Figure 4) and postexercise PON (*r* = 0.6, *p* = 0.0002) (Figure 5). No correlation of HDL-C with PON1c was found.

## 4. Discussion

### 4.1. The Effect of Three Repeated Bouts of Exercise on PON Activity, PON1c, and Other Biochemical Variables in Plasma

The study is firstly aimed at investigating and comparing the effects of three repeated bouts of exercise with a time interval of three days on PON1 activity and concentration. To our best knowledge, this is the first study to explore PON1 response to repeated bouts of exercise in humans.

The primary finding was that a strenuous treadmill run causes an increase in PON and PON1c. This effect is in step with some earlier studies [10, 11, 21]. The exact mechanism by which PON1 is secreted to plasma in acute exercise is yet to be explained. PON1 is expressed on the cell surface of hepatocyte cell lines and requires HDL for secretion [22]. Apolipoprotein A-I and phospholipids present on HDL particles are thought to play a role in the release and regulation of activity and stability of the enzyme [23]. Training and acute exercise were found to modify the phospholipid fatty acid composition [24], probably adding to the effect of PON1 secretion. In the current study, PON comes back to its basal level after three days. Our previous observations show that the return of PON to the basal level occurs much sooner, even two hours after exercise [10]. However, certain changes in PON are induced by repeating the strenuous exercise. We found that with each subsequent exercise session, the percentage increment of PON was higher. Therefore, the protective role of PON1 against oxidative stress is increasing, which suggests the development of an adaptation mechanism to repeated bouts of acute exercise. This may be achieved through the upregulation of redox-sensitive gene expression and antioxidant enzyme levels [25], an increase in enzyme activity [26], stimulation of protein turnover [27], and improvement in DNA-repair systems [28]. In the long run, these mechanisms could potentially lead to increased PON1 activity in sportsmen. Baghaiee et al. described an increase in plasma PON1 activity after 4, 8, and 12 weeks of aerobic training in hypertensive men [29]. Similarly, Senti et al. found that physically active subjects had a higher PON activity than a sedentary group [30]. This implies that there are some mechanisms, which activated by physical activity, improve PON1. Another study, however, did not prove these results [31]. The conflicting results may be due to a different training period in these investigations.

PON1c also increased after each treadmill run. What is more, the increments in PON1c are the highest sooner, already after the second bout of exercise, suggesting that the adaptive mechanisms to increased oxidative stress cause an increase in protein release faster than enzyme activation. Moreover, the increments in PON1c are much higher than the increments in PON. After standardizing PON for PON1c, we found that the PON/PON1c ratio is repeatedly lower postexercise in comparison to preexercise. Even though PON activity is increasing with bouts of exercise, it seems to prominently be an effect of the greater increment in PON1c. We speculate that although more enzyme is released to the circulation, and some of its activity is continually being used up to counteract the deleterious effects of oxidative stress during the acute exercise bout. The enzyme hydrolyzes lipid peroxides and hydroperoxides in oxidized HDL and LDL [32]. Furthermore, its activity may itself be impaired by ROS. PON may be reduced in the course of acute exercise due to the oxidation of PON1's active center's cysteine residues, as its sulfhydryl groups are redox-sensitive [33]. However, repetitions of exercise, if conducted regularly, enhance the antioxidant defence system by releasing increased amounts of free radicals, which may upregulate antioxidant enzyme expression [27].

The increment in PON1 was accompanied by an increase in FRAP. The increase in markers of antioxidant status is usually observed in response to acute exercise [10–12, 34]. FRAP increment was the same in all three bouts of exercise. The level of FRAP depends on many antioxidants, not only enzymatic. An antioxidant, which can bust the total antioxidant capacity of plasma during strenuous exercise, is uric acid. It is released from the cells due to increased catabolism of purine nucleotides. We did not measure it in this study, yet our previous observations confirm that FRAP increments at exercise are, to a large degree, dependent on uric acid formation [10–12]. 72 hours after each exercise, FRAP returned to basal levels. In our previous studies, we have found that FRAP was still increasing 2 hours after exercise [10–12]. We do not know when FRAP values cease to increase further. Yet, in this study, we found that 72 hours after exercise, FRAP comes back to its resting state. We hypothesize that the return of FRAP to baseline values may also depend on the type, intensity, and time of exercise.

### 4.2. Correlation between PON and Other Parameters

Correlations of PON1 activity and factors measured at exercise show that pre- and postexercise PON activity strongly depends on HDL-C concentration. This association can be explained by the linkage of PON1 to HDL-C molecule by Apo A-I. Therefore, changes in HDL-C concentration may lead to similar changes in PON activity. However, in some studies, the PON to HDL-C correlation at exercise is not as strong [10], and in other studies, it is not found [21]. This may be influenced by PON1 Q192R and L55M polymorphism. Additionally, there are other factors, which influence PON1 activity apart from HDL-C concentration. The oxidant changes of unsaturated fatty acids in HDL phospholipids, triglycerides, and apolipoproteins, especially Apo A-I, may modify the HDL particle and change its effect on PON1 enzyme. Moreover, during exercise, the plasma pH decreases, which can influence the PON1 active center and modify the enzyme activity independently of HDL–C concentration and even PON1c. The optimal pH for the activity of PON1 towards both substrates is 7.5–8 [35]. The physiological pH is below this level; therefore, it seems logical to assume that even a slight and transitory fall in pH taking place during strenuous exercise could negatively affect the kinetic performance of the enzyme.

It was of our interest to study the effect of lactate produced at high intensities of exercise on PON1. We found a positive correlation between lactate concentration and PON. This observation is supported by the study of *Sureda* et al. They found that higher lactate concentration during exercise coincided with increased PON during exercise in hyperthermia in comparison to normal temperature conditions [36]. However, the authors do not describe a correlation between these two factors. Further studies on this correlation are warranted.

As we mentioned earlier, also uric acid concentration increases at exercise, having a great effect on the total antioxidant capacity of plasma. Some studies indicate that there may be a negative association between uric acid and PON1 activity [37, 38]. We speculate that the increase in uric acid concentration at exercise may be part of the reason the correlation between preexercise PON activity and concentration ceased to exist postexercise.

An interesting finding of this study is the correlation of preexercise PON with VO_2_max, indicating that subjects with a higher cardiovascular fitness level may have a higher resting level of PON. In our previous study, we have found an association between PON1 arylesterase activity after maximal exercise and the combination of maximal HR and VO_2_max/HR, proving some but not strong association between physical fitness and PON1 activity [10]. Various studies have shown that physically active subjects have a higher resting PON1 activity than sedentary controls [30, 39]. Sedentary groups enrolled in aerobic training protocols were found to have an increase in PON1 activity [29]. One would suspect that also their VO_2_max increased after such intervention. It would be valuable to check their cardiovascular fitness indices before and after such intervention and correlate them with PON1 activity to further investigate this matter.

Another finding of this study is that longer running distance and time of exercise resulted in lower increases of postexercise PON. This observation is in line with previous reports. While experiments with shorter exercise time and running distance lead to increases in postexercise PON [10–12, 21], no change in PON was described after a half-marathon and marathon run [40]. This indicates that the antioxidant defence provided by PON may not last for a long time, and when physical activity was overextended, some of the enzyme activity may have been consumed.

Endurance training includes exercise sets similar to those included in our study. Therefore, our findings provide an experimental proof of endurance training adaptation in antioxidant enzymes assessed by changes in PON1 concentration and activity.

### 4.3. The Study Limitations

The major limitation of this study is that it was conducted on a small number of subjects. We have planned the sample size based on the experiments of similar design, which we and others had conducted previously [11, 24, 34, 41]. For a single comparison before vs. at the bout of acute exercise for the sample size *N* = 11, the estimated effect size was 1, power = 0.8, alpha value = 0.05, and correlation of results was approximately 0.6. Similarly as in our previous studies, when planning the experiment, we have focused on exposing relatively large effects. The limitation of the study is that with the small sample size, the false discovery rate may potentially be higher than 5%. Yet, the presented results are those of preliminary study, and we do not attempt to draw strong conclusions from them.

It was also not possible to investigate the effect of PON1 polymorphisms on the antioxidant defence provided by PON1. A larger sample size to ensure a representative distribution of PON1 polymorphisms among participants should be considered in the future research.

### 4.4. Overview

1. We observed that the exercise resulted in PON increments, regularly increasing after each subsequent repetition
2. PON1c increased, and the value of PON/PON1c decreased each time at the bout of exercise
3. After analysis of the correlations, we speculate that subjects with higher cardiovascular fitness levels, measured as VO_2_max, elicit higher basal levels of HDL-C, PON1c, and PON
4. The positive correlation of postexercise PON with the lactate level confirms the dependence of PON on a load of exercise
5. The time and distance of exercise negatively correlate with the level of PON increase at the bout of exercise, which may be a result of the utilization of PON1 in the antioxidant process

## 5. Conclusions

We postulate that the observed increase in PON and PON1c accompanied by a rise in HDL-C and FRAP at an acute bouts of strenuous aerobic exercise is one of the mechanisms underlying the beneficial effects of physical activity in the prevention of atherosclerosis. We had observed this effect of a single acute exercise in our previous research, yet in this study, we have found that it occurs regularly in each repetition of an exercise bout. Therefore, we conclude that PON1 does not show tolerance to physical activity. Thus, these results suggest that aerobic exercise, if practiced regularly, may evoke intrinsic antioxidant defence mechanisms and lead to a more favourable lipid profile.

Another finding of our study is that a higher cardiovascular fitness level of subjects, assessed as VO_2_max, may be associated with increased PON at rest.

The results of previous studies on the effect of exercise on PON1 status were not always consistent. The underlying mechanisms of these changes should be further investigated, as they may serve as a potential intervention to improve cardiovascular health.

## Figures and Tables

**Table 1:** The sequences of VIC and FAM probes used for polymorphism detection.

Gene	Polymorphism	Nucleotide base	Context sequence [VIC/FAM]
PON1	rs662	C/T	TAAACCCAAATACATCTCCCAGGAT[C/T] GTAAGTAGGGGTCAAGAAAATAGTG
PON1	rs854560	A/T	GCCAGTCCATTAGGCAGTATCTCCA[A/T] GTCTTCAGAGCCAGTTTCTGCCAGA

**Table 2:** Parameters monitored during three bouts of exercise (mean ± SD).

Parameter	Bouts of exercise		
	1st bout	2nd bout	3rd bout
Resting HR (beats/min)	72 ± 11	76 ± 12	69 ± 9
Maximal HR at bout (beats/min)	184 ± 10	183 ± 11	176 ± 12^∗^^#^
BP at rest (mmHg)	127 ± 6/80 ± 4	128 ± 7/80 ± 6	122 ± 9/74 ± 5^#^
BP at the bout (mmHg)	172 ± 20/82 ± 11	169 ± 13/80 ± 10	166 ± 13/79 ± 11
Running distance (km)	8.6 ± 5.5	10.7 ± 7.6^∗^	10.4 ± 7.2^∗^
Running time (min)	47 ± 31	57 ± 41^∗^	56 ± 40^∗^

HR: heart rate; BP: blood pressure. ^∗^vs. corresponding value at 1st bout, *p* < 0.05. ^#^vs. corresponding value at 2nd bout, *p* < 0.05.

**Table 3:** Biochemical measurements in blood (mean ± SD).

Biochemical measurements	First exercise		Second exercise		Third exercise	
	Before	Bout	Before	Bout	Before	Bout
PON (U/L)	884.4 ± 96.2	941.8^∗^ ± 93.1	831 ± 83.8	914.6^∗^ ± 98.8	858.2 ± 83.5	964^∗^ ± 86.8
PON1c (*μ*g/mL)	1.62 ± 0.14	2.67^∗^ ± 0.2	1.55 ± 0.11	2.99^∗^ ± 0.25	1.61 ± 0.1	2.85^∗^ ± 0.26
FRAP (mM/L Fe^+2^)	1.15 ± 0.06	1.34^∗^ ± 0.07	1.07 ± 0.05	1.29^∗^ ± 0.06	1.18 ± 0.05	1.32^∗^ ± 0.04
TChol (mM/L)	5.64^#^ ± 0.26	6.15^∗^ ± 0.3	5.4 ± 0.28	5.54 ± 0.43	5.35 ± 0.2	5.78^∗^ ± 0.19
HDL-C (mM/L)	1.34 ± 0.05	1.45^∗#^ ± 0.05	1.29 ± 0.05	1.39^∗^ ± 0.06	1.32 ± 0.04	1.4^∗^ ± 0.05
LDL-C (mM/L)	3.42^#^ ± 0.25	3.73^∗#^ ± 0.27	3.47 ± 0.27	3.61 ± 0.23	3.26 ± 0.17	3.49^∗^ ± 0.16
TG (mM/L)	1.99^#^ ± 0.27	2.15 ± 0.32	1.43 ± 0.17	1.86^∗^ ± 0.23	1.71 ± 0.23	1.93 ± 0.22
PON/HDL-C	653.0 ± 62.1	644.7 ± 56.9	641.9 ± 57.4	653.7 ± 65.8	648.6 ± 58.4	687.1 ± 56.4
PON/PON1c	569.6 ± 70.7	374.5^∗^ ± 47.6	539.8 ± 45.3	319.3^∗^ ± 35.7	540.1 ± 54.7	364.2^∗^ ± 45
Lactate (mM/L)	1.72 ± 0.24	8.95^∗^ ± 1.46	1.42 ± 0.21	8.83^∗^ ± 1.59	1.56 ± 0.15	7.97^∗^ ± 1.42
Creatinine (*μ*M/L)	84.82 ± 3.67	116.0^∗#^ ± 2.16	84.27 ± 3.17	113.55^∗^ ± 3.51	85.82 ± 4.38	105.91^∗^ ± 3.99
Urea (mM/L)	5.82^#^ ± 0.37	6.43^∗^ ± 0.48	5.73 ± 0.35	6.36^∗^ ± 0.45	6.13 ± 0.53	6.69^∗^ ± 0.54
CK (U/L)	162.2^#^ ± 20.1	210.8^∗#^ ± 35.5	266.6 ± 68.4	301.8 ± 58.3	300.8 ± 41.7	348.4^∗^ ± 41.0
CRP (mg/L)	0.99 ± 0.19	1.53^∗^ ± 0.41	1.31 ± 0.35	1.29 ± 0.41	1.73 ± 0.56	1.92 ± 0.60

PON: paraoxonase activity; PON1c: paraoxonase 1 concentration; FRAP: ferric-reducing activity of plasma; TChol: total cholesterol; HDL-C: high-density lipoprotein cholesterol; LDL-C: low-density lipoprotein cholesterol; TG: triglyceride; CK: creatine kinase; CRP: C-reactive protein. ^∗^Before vs. bout of exercise, *p* < 0.05. ^#^First vs. second and third exercise, *p* < 0.05.

## Data Availability

The data used to support the findings of this study are available from the corresponding author upon request.

## Supplementary Materials

Supplementary Materials Table S1: individual data of pre- and postexercise paraoxonase activity (PON) in average-trained men at each of the three bouts of strenuous exercise. Table S2: individual data of pre- and postexercise paraoxonase 1 concentration (PON1c) in average-trained men at each of the three bouts of strenuous exercise. Table S3: individual data of pre- and postexercise ferric-reducing activity of plasma (FRAP) in average-trained men at each of the three bouts of strenuous exercise. Table S4: individual data of pre- and postexercise high-density lipoprotein concentration (HDL-C) in average-trained men at each of the three bouts of strenuous exercise. Table S5: PON1 genotype distribution among participants by individual cases. Table S6: ANOVA—complete summary of the repeated measures ANOVA of biochemical measurements for the three repeated exercise bouts. Table S7: the ANOVA Friedman test and Kendall's coefficient of concordance of the biochemical measurements in the three repeated exercise bouts. Table S8: resulting *p* values of the Student *t*-test/Wilcoxon pairwise comparisons between before vs. at the bout of exercise in each repetition of exercise. Figure S1: scatterplot of variables *x* and *y*. Correlation of postexercise values of PON and lactate concentration in eleven average-trained healthy men. Pooled individual postexercise data from three exercise bouts (*n* = 33; *r* = 0.44, *p* = 0.01).
