# Supplementary material for: Repetitions of Strenuous Exercise Consistently Increase Paraoxonase 1 Concentration and Activity in Plasma of Average-Trained Men
Source: Oxid Med Cell Longev. 2021 Dec 7;2021:2775025. doi: 10.1155/2021/2775025 (PMC8670925; doi:10.1155/2021/2775025)
Supplement: Supplementary Materials — Table S1: individual data of pre- and postexercise paraoxonase activity (PON) in average-trained men at each of the three bouts of strenuous exercise. Table S2: individual data of pre- and postexercise paraoxonase 1 concentration (PON1c) in average-trained men at each of the three bouts of strenuous exercise. Table S3: individual data of pre- and postexercise ferric-reducing activity of plasma (FRAP) in average-trained men at each of the three bouts of strenuous exercise. Table S4: individual data of pre- and postexercise high-density lipoprotein concentration (HDL-C) in average-trained men at each of the three bouts of strenuous exercise. Table S5: PON1 genotype distribution among participants by individual cases. Table S6: ANOVA—complete summary of the repeated measures ANOVA of biochemical measurements for the three repeated exercise bouts. Table S7: the ANOVA Friedman test and Kendall's coefficient of concordance of the biochemical measurements in the three repeated exercise bouts. Table S8: resulting p values of the Student t-test/Wilcoxon pairwise comparisons between before vs. at the bout of exercise in each repetition of exercise. Figure S1: scatterplot of variables x and y. Correlation of postexercise values of PON and lactate concentration in eleven average-trained healthy men. Pooled individual postexercise data from three exercise bouts (n = 33; r = 0.44, p = 0.01). [file 2775025.f1.pdf]

Supplementary material for

Repetitions of strenuous exercise consistently increase paraoxonase 1 concentration and activity in plasma of average-trained men

Aneta Otocka-Kmiecik, Monika Orłowska-Majdak, Robert Stawski, Urszula Szkudlarek, Piotr Kosielski, Gianluca Padula, Szymon Gałczyński, Dariusz Nowak

Supplementary Table S1. Individual data of pre- and post-exercise paraoxonase activity (PON) in average-trained men at each of the three bouts of strenuous exercise.

	Participant number	1st bout		2nd bout		3rd bout	
		**Pre-exercise**	**Post-exercise**	**Pre-exercise**	**Post-exercise**	**Pre-exercise**	**Post-exercise**
**PON (U/L)**	1	343,5	469,5	443,2	472,3	466,8	479,2
	2	1102,5	1110,8	825,5	871,2	1091,4	1059,6
	3	804,7	982,0	825,5	871,2	785,3	896,1
	4	403,0	443,2	421,1	430,7	436,3	482,0
	5	1156,5	1188,4	1116,3	1404,4	1060,9	1350,4
	6	520,8	527,7	475,1	533,2	452,9	818,6
	7	1023,5	1146,8	1015,2	1121,9	994,5	1128,8
	8	1148,2	1127,4	958,4	1070,6	1029,1	984,8
	9	1081,7	1246,5	910,0	1198,1	1062,3	1221,6
	10	1206,4	1166,2	1261,8	1236,8	1133,0	1250,7
	11	937,7	951,5	889,2	850,4	928,0	932,1

Supplementary Table S2. Individual data of pre- and post-exercise Paraoxonase 1 concentration (PON1c) in average-trained men at each of the three bouts of strenuous exercise.

	Participant number	1st bout		2nd bout		3rd bout	
		**Pre-exercise**	**Post-exercise**	**Pre-exercise**	**Post-exercise**	**Pre-exercise**	**Post-exercise**
**PON1C (µg/mL)**	1	1,55	2,95	1,28	1,99	1,32	2,21
	2	1,19	2,15	1,14	2,22	1,24	2,2
	3	1,42	2,86	1,3	3,42	1,39	3,59
	4	1,46	2,7	1,56	3,67	1,64	2,91
	5	1,62	2,66	2,04	3,33	1,93	3,92
	6	1,21	2,03	1,15	1,99	1,31	1,66
	7	1,51	1,96	1,62	2,43	1,68	2,58
	8	1,29	3,15	1,28	3,4	1,36	3,97
	9	1,74	2,42	1,57	2,46	1,65	2,13
	10	1,98	2,12	2,24	3,31	2,21	2,23
	11	2,83	4,31	1,84	4,63	1,95	3,96

Supplementary Table S3. Individual data of pre- and post-exercise ferric reducing activity of plasma (FRAP) in average-trained men at each of the three bouts of strenuous exercise.

	Participant number	1st bout		2nd bout		3rd bout	
		**Pre-exercise**	**Post-exercise**	**Pre-exercise**	**Post-exercise**	**Pre-exercise**	**Post-exercise**
**FRAP (mM/L Fe^{+2})**	1	92,7	63,7	89,1	90,7	57,5	91,2
	2	153,9	130,6	234,2	242,5	160,1	160,6
	3	123,3	187,0	177,7	188,1	143,5	171,0
	4	166,3	128,5	152,3	134,7	126,4	81,9
	5	224,9	165,8	218,7	239,4	174,1	178,2
	6	177,2	229,5	305,7	190,7	104,7	232,1
	7	117,1	211,9	134,7	161,1	132,1	148,2
	8	120,7	164,2	65,3	159,6	147,7	135,2
	9	256,5	167,9	166,8	97,4	160,1	139,9
	10	157,0	114,0	132,1	82,9	85,0	79,3
	11	92,2	67,9	86,5	50,8	97,9	77,2

Supplementary Table S4. Individual data of pre- and post-exercise high-density lipoprotein concentration (HDL -C) in average-trained men at each of the three bouts of strenuous exercise.

	Participant number	1st bout		2nd bout		3rd bout	
		**Pre-exercise**	**Post-exercise**	**Pre-exercise**	**Post-exercise**	**Pre-exercise**	**Post-exercise**
**HDL-C (mM/L)**	1	1,21	1,26	1,14	1,2	1,3	1,39
	2	1,31	1,41	1,35	1,34	1,25	1,25
	3	1,14	1,3	1,14	1,24	1,18	1,22
	4	1,14	1,22	1,16	1,26	1,23	1,39
	5	1,55	1,63	1,23	1,37	1,48	1,62
	6	1,33	1,52	1,36	1,47	1,24	1,33
	7	1,32	1,4	1,26	1,32	1,32	1,27
	8	1,34	1,5	1,29	1,53	1,26	1,41
	9	1,43	1,53	1,27	1,38	1,38	1,47
	10	1,7	1,82	1,76	1,88	1,62	1,76
	11	1,23	1,34	1,21	1,33	1,22	1,32

Supplementary Table S5. PON1 genotype distribution among participants by individual cases.

Participant number	Q192R polymorphism	L55M polymorphism
1	QQ	LL
2	QR	LM
3	QR	LM
4	QQ	LM
5	QR	LL
6	QQ	LM
7	QR	LL
8	QR	LM
9	QR	LM
10	RR	LL
11	QR	LL

Supplementary Table S6. ANOVA – complete summary of the repeated measures ANOVAs analysis of biochemical measurements for the three repeated exercise bouts, when normal data distribution was found

**Compared biochemical measurements**	**ANOVA parameters**			
	**DF**	**MS (variance)**	**F**	**p**
**PON bout (U/L)**	0,5744	0,2872	1,7192	0,204659
**PON1c bout (µg/mL)**	0,5744	0,2872	1,7192	0,204659
**FRAP before (mM/L Fe^{+2})**	0,06698	0,03349	2,4179	0,114684
**FRAP bout (mM/L Fe^{+2})**	0,01723	0,00862	0,4733	0,629731
**TChol before (mM/L)**	0,5260	0,2630	2,5309	0,104757
**TChol bout (mM/L)**	2,112	1,056	2,2061	0,136213
**HDL-C before (mM/L)**	0,01289	0,00645	1,2382	0,311192
**LDL-C before (mM/L)**	0,2711	0,1355	1,1608	0,333475
**LDL-C bout (mM/L)**	0,3266	0,1633	1,7996	0,191128
**PON/PON1c before**	6446	3223	0,71343	0,502014
**PON/PON1c bout**	18928	9464	2,19029	0,137994
**Lactate before (mM/L)**	0,51139	0,25569	1,09895	0,352517
**Lactate bout (mM/L)**	6,227	3,114	1,83854	0,184935
**Creatinine before (µM/L)**	13,5	6,8	0,3923	0,680567
**Creatinine bout (µM/L)**	609,3	304,6	3,723	0,042234
**Urea before (mM/L)**	0,972	0,486	0,7744	0,474302

DF – degrees of freedom, MS – mean square, F - distribution value, p – probability, SD – standard deviation, PON – paraoxonase activity, PON1c – paraoxonase 1 concentration, FRAP – ferric reducing activity of plasma, TChol – total cholesterol, HDL-C – high density lipoprotein cholesterol, LDL-C - low density lipoprotein cholesterol, TG – triglicerides, CK – creatine kinase, CRP – C-reactive protein

Supplementary Table S7. The ANOVA Friedman test and Kendall's coefficient of concordance of the biochemical measurements in the three repeated exercise bouts, when the data do not follow a normal distribution

**Compared biochemical measurements**	**χ2 ANOVA**	**τ-Kendall**	**p**
**PON before (U/L)**	3.45	0.16	0.178
**PON1c before (µg/mL)**	4.55	0.21	0.103
**HDL-C bout (mM/L)**	5.64	0.26	0,05
**TG before (mM/L)**	7.09	0.32	0,029
**TG bout (mM/L)**	4.91	0.22	0.086
**PON/HDL-C before**	0.55	0.03	0.761
**PON/HDL-C bout**	0.55	0.03	0.761
**Urea bout (mM/L)**	0.18	0.01	0.913
**CK before (U/L)**	9.45	0.43	0.009
**CK bout (U/L)**	8.91	0.405	0.012
**CRP before (mg/L)**	0.65	0.03	0.723
**CRP bout (mg/L)**	0.24	0.01	0.24

χ2 ANOVA - Chi square critical value, τ-Kendall - *Kendall* rank correlation coefficient, p – probability, PON – paraoxonase activity, PON1c – paraoxonase 1 concentration, HDL-C – high density lipoprotein cholesterol, TG – triglicerides, CK – creatine kinase, CRP – C-reactive protein

Supplementary Table S8. Resulting p values of the Student's t-test/Wilcoxon pairwise comparisons between before vs at the bout of exercise in each repetition of exercise

**Compared biochemical measurements**	**t-test**			**Wilcoxon test**		
	**1st bout**	**2nd bout**	**3rd bout**	**1st bout**	**2nd bout**	**3rd bout**
**PON (U/L)**	-	0.031	-	0.040	-	0.020
**PON1c (µg/mL)**	-	-	0.000	0.003	0.003	-
**FRAP (mM/L Fe^{+2})**	0.033	0.000	0.000	-	-	-
**TChol (mM/L)**	0.001	0.649	0.000	-	-	-
**HDL-C (mM/L)**	0.000	-	-	-	0.004	0.009
**LDL-C (mM/L)**	0.019	0.242	0.001	-	-	-
**TG (mM/L)**	0.108	-	-	-	0.007	0.059
**PON/HDL-C**	-	0.606	-	0.593	-	0.328
**PON/PON1c**	0.001	0.000	0.011	-	-	-
**Lactate (mM/L)**	0.000	0.000	0.001	-	-	-
**Creatinine (µM/L)**	0.000	0.000	0.001	-	-	-
**Urea (mM/L)**	0.000	0.002	-	-	-	0.007
**CK (U/L)**	-	-	0.006	0.032	0.051	-
**CRP (mg/L)**	-	-	-	0.005	0.916	0.139

PON – paraoxonase activity, PON1c – paraoxonase 1 concentration, FRAP – ferric reducing activity of plasma, TChol – total cholesterol, HDL-C – high density lipoprotein cholesterol, LDL-C - low density lipoprotein cholesterol, TG – triglicerides, CK – creatine kinase, CRP – C-reactive protein

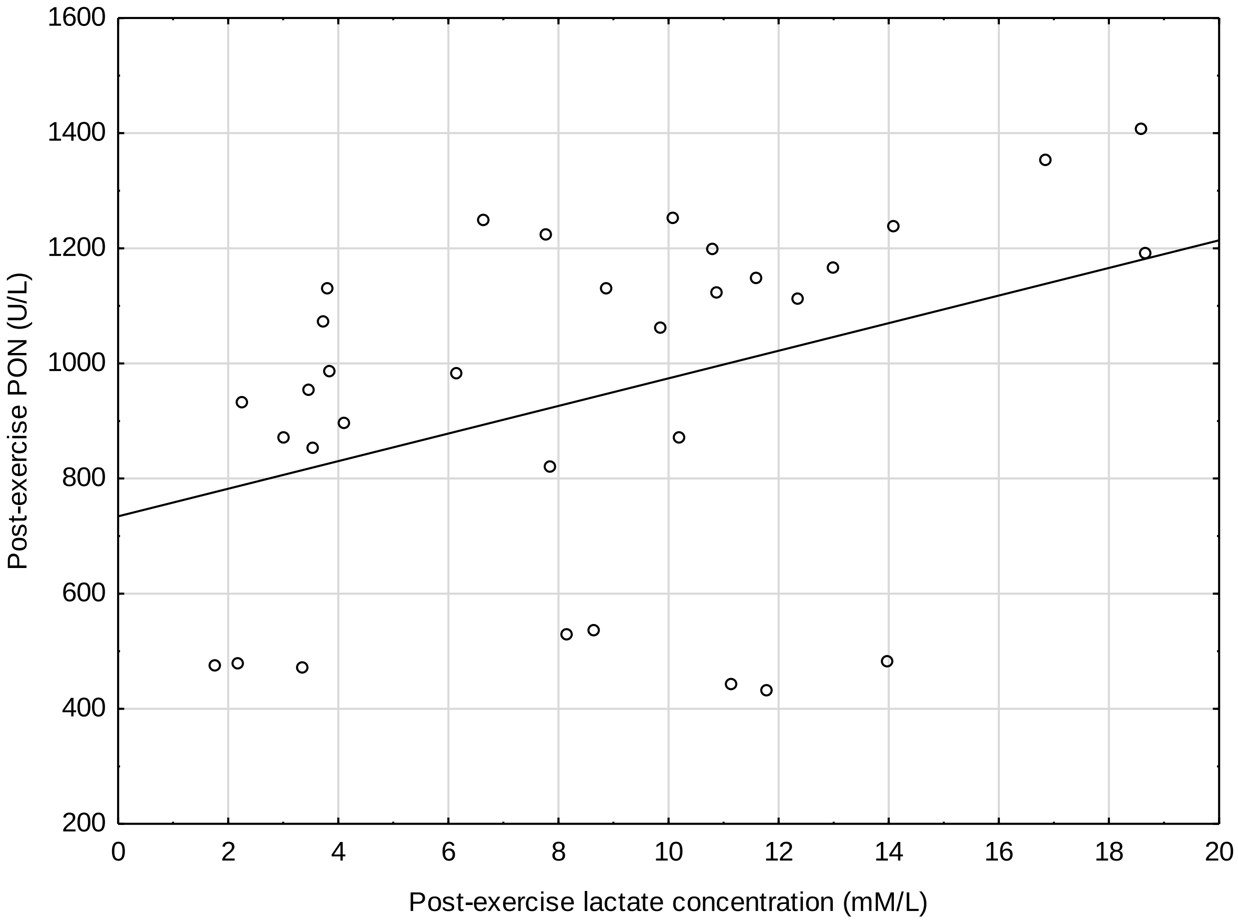

Supplementary Figure S1. Scatterplot of variables x and y. Correlation of post-exercise values of PON and lactate concentration in eleven average-trained healthy men. Pooled individual post-exercise data from three exercise bouts (n=33; r=0.44, p=0.01).
